# Modeling of multivariate longitudinal phenotypes in family genetic studies with Bayesian multiplicity adjustment

**DOI:** 10.1186/1753-6561-8-S1-S69

**Published:** 2014-06-17

**Authors:** Lili Ding, Brad G Kurowski, Hua He, Eileen S Alexander, Tesfaye B Mersha, David W Fardo, Xue Zhang, Valentina V Pilipenko, Leah Kottyan, Lisa J Martin

**Affiliations:** 1 Department of Pediatrics, Cincinnati Children's Hospital Medical Center, 3333 Burnet Avenue, Cincinnati, OH 45229, USA; 2 Department of Pediatrics, University of Cincinnati College of Medicine, 3235 Eden Avenue,Cincinnati, OH 45267, USA; 3 Department of Environmental Health, University of Cincinnati College of Medicine, 3223 Eden Avenue, Cincinnati, OH 45267, USA; 4 Department of Biostatistics, University of Kentucky, 725 Rose Street, Lexington, KY 40536, USA

## Abstract

Genetic studies often collect data on multiple traits. Most genetic association analyses, however, consider traits separately and ignore potential correlation among traits, partially because of difficulties in statistical modeling of multivariate outcomes. When multiple traits are measured in a pedigree longitudinally, additional challenges arise because in addition to correlation between traits, a trait is often correlated with its own measures over time and with measurements of other family members. We developed a Bayesian model for analysis of bivariate quantitative traits measured longitudinally in family genetic studies. For a given trait, family-specific and subject-specific random effects account for correlation among family members and repeated measures, respectively. Correlation between traits is introduced by incorporating multivariate random effects and allowing time-specific trait residuals to correlate as in seemingly unrelated regressions. The proposed model can examine multiple single-nucleotide variations simultaneously, as well as incorporate familyspecific, subject-specific, or time-varying covariates. Bayesian multiplicity technique is used to effectively control false positives. Genetic Analysis Workshop 18 simulated data illustrate the proposed approach's applicability in modeling longitudinal multivariate outcomes in family genetic association studies.

## Background

High-throughput genotyping advances have generated large amounts of genetic data. At the same time, numerous related phenotypes are often collected in genetic studies of complex traits. To understand how genetic variants influence multiple traits, it is necessary to consider correlation among variants and multiple traits jointly [1,2]. However, most genetic studies have focused on single variant-single trait association. Analysis performed in this fashion faces at least 3 issues. First, it leads to concerns regarding false discoveries. Bonferroni andother types of correction may limit false discoveries, but implementing these methods on correlated traits limits the power to detect true associations. Second, same genetic variants may influence multiple related traits [3]. Thus, modeling traits separately may misrepresent the underlying biology. Lastly, failure to integrate correlation among variants because of linkage disequilibrium (LD) dilutes the association. Longitudinal studies provide an additional challenge because traits measured over time are likely to be correlated.

We propose a Bayesian joint modeling of longitudinally measured multiple traits in family genetic studies. Explicitly considering the correlation structure between multiple traits using multivariate random effects [4] and seemingly unrelated regression techniques [5], this model studies simultaneously multiple single-nucleotide variations (SNVs) and their associations with multiple traits measured longitudinally. Bayesian multiplicity [6] correction controls false positives. Our method uniquely demonstrates the ability of Bayesian methods to account for the complexity of multivariate, longitudinal data in the context of genetic family data while controlling for false positives. Simulated data from Genetic Analysis Workshop 18 (GAW18) illustrate the application of the proposed method. The analysis was performed with knowledge of the simulation model.

## Methods

### Bayesian bivariate model

We consider SNV association models with a longitudinal bivariate quantitative outcome in family studies. Let $y_{ijt}^{k}$ be the $k^{th}$ outcome of subject *j *of pedigree *i *at time *t*. Here k=1 or 2 for bivariate outcomes; i=1,2,…,I indexes *I *families in the study; $j=1,2,\ldots ,n_{i}$ indexes $n_{i}$ subjects in pedigree *i*; and $t=1,2,\ldots m_{ij}$ indexes time points where outcomes are measured for subject *j *of pedigree *i*. Here, the number of measurement $m_{ij}$ does not need to be equal for different subjects, but we assume the 2 outcomes are measured at the same set of time points for the same individual. We jointly model the outcomes as follows:

$$y_{ijt}^{k}=\beta_{0}^{k}+X_{ij}^{T}\beta_{x}^{k}+Z_{ijt}^{T}\beta_{z}^{k}+{\text{Σ}}_{g=1}^{G}\gamma_{g}^{k}\beta_{g}^{k}SNV_{ijg}+p_{i}^{k}+s_{ij}^{k}+e_{ijt}^{k}$$

Here, *X *is a vector of subject-specific time-invariant covariates and *Z *is a vector of time-varying covariates. $SNV_{ijg},g=1,2,\ldots G,$ is the $g^{th}$ SNV of subject *j *of pedigree *i*, coded as 0, 1, or 2 indicating the number of minor alleles and *G *is the number of SNVs in the model. $\beta^{\prime }\text{s}$ are regression coefficients (including an intercept) for the covariates or genetic variants. $\gamma^{\prime }\text{s}$ are SNV-specific indicator variables, which take values 1 or 0, depending on whether the SNV has an effect on the given outcome or not. Let $p_{i}=\left\{p_{i}^{1},p_{i}^{2}\right\}$, a pedigree-specific random effect common to all the individuals in the same family and accounts for correlation within families. Let $s_{ij}=\left\{s_{ij}^{1},s_{ij}^{2}\right\}$, a subject-specific random effect accounting for the correlation between measures taken repeatedly on the same subject. Let $e_{ijt}=\left\{e_{ijt}^{1},e_{ijt}^{2}\right\},$ the residual error. Additionally, $p_{i}$, $s_{ij}$ and $e_{ijt}$ are assumed to be independent, and

(1) $$\text{Random family}:\;p_{i}\sim N_{2}\left(0,{\text{Σ}}_{p}\right),{\text{Σ}}_{p}=\left(\begin{array}{cc} \sigma_{p_{1}}^{2} & \rho_{p}\sigma_{p_{1}}\sigma_{p_{2}}\\ \rho_{p}\sigma_{p_{1}}\sigma_{p_{2}} & \sigma_{p_{2}}^{2} \end{array}\right);$$

(2) $$\text{Random subject}:\;s_{ij}\sim N_{2}\left(0,{\text{Σ}}_{s}\right),{\text{Σ}}_{s}=\left(\begin{array}{cc} \sigma_{s_{1}}^{2} & \rho_{s}\sigma_{s_{1}}\sigma_{s_{2}}\\ \rho_{s}\sigma_{s_{1}}\sigma_{s_{2}} & \sigma_{s_{2}}^{2} \end{array}\right);$$

(3) $$\text{Residual error}:\;e_{ijt}\sim N_{2}\left(0,{\text{Σ}}_{e}\right),{\text{Σ}}_{e}=\left(\begin{array}{cc} \sigma_{e_{1}}^{2} & \rho_{e}\sigma_{e_{1}}\sigma_{e_{2}}\\ \rho_{e}\sigma_{e_{1}}\sigma_{e_{2}} & \sigma_{e_{2}}^{2} \end{array}\right).$$

This Bayesian bivariate model uses bivariate normal random family and subject effects, and a bivariate normal distribution for the residual errors, as in seemingly unrelated regressions, to jointly model the 2 traits and allow them to be correlated.

### Bayesian multiplicity adjustment

Bayesian multiplicity adjustment is a Bayesian solution to multiplicity problems [6]. Through appropriate choices of priors on model probabilities, this Bayesian solution will not inflate type I error in the face of multiple comparisons. In our model, we use prior distributions on SNV inclusion to provide correction for multiplicity. For each SNV in the model, 2 parameters are present--the inclusion indicator and the regression coefficient. We assume that the inclusion indicators follow a Bernoulli distribution with an unknown inclusion probability *q*, while *q *follows a Beta distribution, that is, $\gamma_{g}^{k}\sim Bernoulli\left(1,q\right)$ and $q\sim Beta\left(a,b\right)$. Here, we set a=1,b=G, which represents a sceptical prior such that the marginal prior odds of an association is 1/G[7]. This Beta-Bernoulli type of prior provides an intrinsic multiplicity correction in the face of multiple comparisons. The significance of the Bayesian analysis is the posterior inclusion probability. Unlike traditional *p* values, a higher posterior inclusion probability indicates greater confidence of a true result. For each SNV, we estimate posterior inclusion probability as

$$\hat{p}\left(\gamma_{g}^{k}=1|D\right)={\text{Σ}}_{M_{\gamma^{k}}\in S}I_{\gamma_{g}^{k}=1}\hat{p}\left(M_{\gamma^{k}}|D,S\right)$$

Here, *S *is the model space being visited by the Markov chain Monte Carlo (MCMC); $\hat{p}\left(M_{\gamma^{k}}|D,S\right)$ is the estimated posterior probability of model $M_{\gamma^{k}}$, where $\gamma^{k}=\left\{\gamma_{1}^{k},\gamma_{2}^{k},\ldots ,\gamma_{G}^{k}\right\}$ is a vector of 0's and 1's, indicating which SNVs are in the model or not. $I_{\gamma_{g}^{k}=1}=1$ if $\gamma_{g}^{k}=1$ and 0 otherwise. True- and false-positive rates are based on the median probability model, which is the model that includes variants with a posterior inclusion probability larger or equal to 0.5. We use noninformative, independent,univariate normal priors for all regression coefficients $\beta_{0}^{k},\beta_{x}^{k},\beta_{z}^{k}$, and $\beta_{g}^{k}$, and noninformative inverse Wishart priors for variance-covariance matrices ${\text{Σ}}_{p},{\text{Σ}}_{s}$, and ${\text{Σ}}_{e}$.

### Analysis of GAW18 data

Two outcomes, diastolic (DBP) and systolic blood pressure (SBP), were evaluated. Covariates included sex, age, and smoking status. To adjust for antihypertensive medications use, we calculated the mean difference of blood pressure (BP) between observations with medication use (med = 1) and those without medication (med = 0) among observations with hypertension (htn = 1);that is, $\left(\bar{BP}|htn=1,med=1)-(\bar{BP}|htn=1,med=0\right)$. Observed BP values were adjusted using mean differences for observations with htn = 1 and med = 1 to impute DBP and SBP measures without medication use. These adjusted outcomes were used in the analysis [8].

Included were 849 individuals from 20 pedigrees (average number of subjects per pedigree was 42; range: 21-75). Each individual has 3 observations. Our focus is on SNVs in *MAP4 *on chromosome 3. There are 894 SNVs in the gene; 14 influence both DBP and SBP, and 1 influences SBP. Among the 15 causal SNVs, three are common (minor allele frequency [MAF]>0.05); all others are rare. All analyses were based on replication set 1. To investigate effectiveness of Bayesian multiplicity adjustment, a random set of noise variants of size 1, 15, 30, 60, and 90 were added from *MAP4*. Selection of noise variants was truly random and did not account for LD with causal SNVs. After burn-in and thinning every 10^th^ iteration, 50,000 samples were drawn for MCMC simulations.

We compared our method with two other methods. One method is a Bayesian univariate model where the off diagonal of the variance and covariance matrices in equations (1), (2), and (3) are zero, thus the 2 traits are modeled independently. The univariate model can handle longitudinal data and multi-variants, and uses Bayesian multiplicity techniques to adjust for multiple comparisons. The other is the family-based measured genotype approach (MGA), which is a standard approach to analyze family genetic studies and compares polygenic models with or without each SNV as a covariate [9]. MGA models a single outcome and single SNV, and cannot handle longitudinal data from multiple outcomes jointly. Thus, only the first pair of DBP and SBP measures, adjusted for medication use, of each individual was used and modeled separately. Bonferroni correction was used for multiple comparisons after accounting for 105 tests (90 noises and 15 causal).

## Results

Using a threshold of 0.5 for the posterior inclusion probability, the Bayesian bivariate model detected 5 of the 15 SNVs between SBP and DBP with 1 noise variant (Table 1). As the number of noise variants increased, the number of true positives identified was reduced, such that by 90 noise variants, only two causal variants were identified. True positives had relatively low MAFs and large effect sizes (Table 1). False negatives either were rare, had small effect size, or both. Importantly, Bayesian multiplicity adjustment yielded no false positives. This suggests that a lower posterior probability threshold may be appropriate to yield a family-wise error rate of 0.05.

**Table 1:** Posterior inclusion probabilities of the causal SNVs

					Number of noise variables													
**Causal SNV**	**Position**	**MAF**			***1***		***15***		***30***		***60***		***90***		90* (UNI)		90* (MGA)	
			
			**D**	**S**	***D***	***S***	***D***	***S***	***D***	***S***	***D***	***S***	***D***	***S***	**D**	**S**	**D**	**S**

1	47912898	0.0049	1.71	2.34	*0.06*	*0.11*	*0.03*	*0.06*	*0.02*	*0.05*	*0.01*	*0.02*	*0.01*	*0.01*	0.03	0.04	2.0E-01	1.7E-01

2	47913455	0.0049	−5.46	−8.70	***0.93***	***0.75***	***0.93***	***0.84***	***0.87***	***0.76***	***0.51***	*0.22*	*0.33*	*0.09*	**0.99**	0.41	6.3E-03	1.4E-02

3	47924216	0.0066	1.35	1.84	*0.03*	*0.04*	*0.02*	*0.02*	*0.01*	*0.01*	*0.01*	*0.01*	*0.00*	*0.00*	0.01	0.01	2.3E-01	4.6E-01

4	47955326	0.0066	−1.93	−2.63	*0.05*	*0.08*	*0.02*	*0.03*	*0.01*	*0.03*	*0.01*	*0.01*	*0.00*	*0.01*	0.01	0.02	5.8E-01	4.6E-01

5	47956424^a^	0.3777	−1.50	−2.38	***0.52***	*0.32*	*0.06*	*0.02*	*0.09*	*0.03*	*0.08*	*0.06*	*0.03*	*0.01*	0.09	0.07	**4.2E-07**	**1.2E-04**

6	47957741	0.0016	−5.08	−8.10	*0.06*	*0.08*	*0.03*	*0.05*	*0.02*	*0.04*	*0.01*	*0.01*	*0.01*	*0.01*	0.01	0.01	9.7E-01	4.4E-01

7	47957996^b^	0.0301	−4.64	−7.39	*0.11*	***0.64***	*0.09*	***0.65***	*0.06*	***0.71***	*0.06*	***0.56***	*0.10*	***0.51***	0.08	0.31	**9.7E-12**	**4.0E-14**

8	47958037^a^	0.3420	0.00	−0.00	*0.22*	*0.33*	*0.06*	*0.06*	*0.05*	*0.06*	*0.02*	*0.05*	*0.02*	*0.02*	0.05	0.08	**3.3E-07**	**4.3E-05**

9	47973345	0.0082	2.14	2.92	*0.08*	***0.58***	*0.04*	*0.47*	*0.03*	*0.42*	*0.02*	*0.19*	*0.01*	*0.12*	0.01	0.09	**1.9E-05**	6.6E-03

10	48040283^b^	0.0318	−6.22	−9.91	***0.97***	***0.52***	***0.95***	*0.45*	***0.96***	*0.36*	***0.96***	*0.46*	***0.91***	***0.51***	**0.94**	**0.71**	**2.6E-13**	**2.3E-14**

11	48040284	0.0131	−6.95	−11.1	*0.42*	*0.38*	*0.12*	*0.08*	*0.08*	*0.07*	*0.05*	*0.04*	*0.03*	*0.02*	0.35	0.26	5.9E-03	2.4E-03

12	48054461	0.1187	0.46	0.63	*0.10*	*0.05*	*0.14*	*0.08*	*0.07*	*0.04*	*0.01*	*0.00*	*0.01*	*0.00*	0.13	0.02	1.4E-02	3.0E-02

13	48061725	0.0050	1.79	2.44	*0.08*	*0.07*	*0.04*	*0.03*	*0.03*	*0.03*	*0.01*	*0.01*	*0.01*	*0.01*	0.02	0.01	9.4E-01	7.4E-01

14	48069438	0.0065	−1.78	−2.43	*0.04*	*0.11*	*0.02*	*0.04*	*0.01*	*0.03*	*0.01*	*0.02*	*0.00*	*0.01*	0.01	0.03	4.2E-01	6.7E-01

15	48091219	0.0065	2.54	3.46	*0.06*	*0.09*	*0.03*	*0.04*	*0.02*	*0.03*	*0.01*	*0.01*	*0.01*	*0.01*	0.01	0.02	7.2E-01	5.6E-01

True positives					*3*	*4*	*2*	*2*	*2*	*2*	*2*	*1*	*1*	*2*	2	1	5	4

False positives					*0*	*0*	*0*	*0*	*0*	*0*	*0*	*0*	*0*	*0*	1	1	8	6

Numbers in italics show the results of the Bayesian bivariate model, that is, estimated posterior inclusion probabilities of the 15 causal SNVs and numbers of true and false positives based on the median probability model. Posterior inclusion probabilities ≥0.5 are in bold. The last 4 columns are the results of the model where DBP and SBP are modeled independently (*) using either the Bayesian univariate model (UNI, posterior inclusion probability) or measured genotype approach (MGA, *p *value), where *p *values in bold are below the cutoff with Bonferroni correction (0.05/105 = 4.76E-04). In the table, *D *represents DBP, *S *represents SBP. SNVs 5 and 8 (^a^) and SNVs 7 and 10 (^b^) have relatively high LD (*r*^2^≥0.8).

Using the same set of 90 noise variants, MGA detected five causal SNVs (4 for SBP and 1 additional for DBP). However, MGA identified 8 and 6 false positives for DBP and SBP, respectively. The Bayesian univariate model identified 2 causal SNVs and 1 false positive for both DBP and SBP.

As the 90 noise variants were randomly selected, examining the LD structure is also important. Among the true positives, two pairs (SNVs 5 and 8 and SNVs 7 and 10) have a high LD $\left(r^{2}\geq 0.8\right)$. SNVs 7 and 10 were both identified by the Bayesian bivariate model and MGA, but only SNV 10 was identified by the Bayesian univariate model. SNVs 5 and 8 were identified by MGA, but none was identified by either the Bayesian bivariate or univariate approach. For MGA, six of the false positives of DBP and four of the false positives of SBP had relatively high LD $\left(r^{2}\geq 0.8\right)$ with identified true positives. After discounting false positives caused by indirect effects, MGA had 2 false positives for both DBP and SBP.

**Figure 1:**
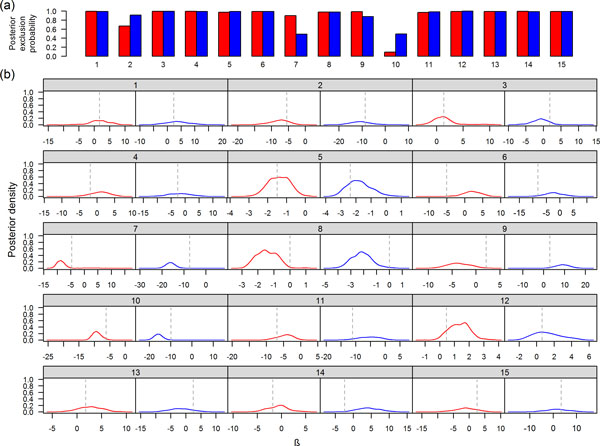
**Posterior exclusion probability and posterior density of regression coefficients of the causal SNVs**. For the Bayesian bivariate model with 90 noise variants, the plot shows, for the 15 causal SNVs, (a) estimated posterior exclusion probabilities (red for DBP and blue for SDP), and (b) posterior density of regression coefficients (red for DBP and blue for SBP) when the SNVs were included in the model. Dashed reference lines indicate simulated effect sizes.

Overall the Bayesian bivariate model resulted in posterior estimates of beta values very similar to the reported effect size from Genetic Analysis Workshop (GAW) data generators (Figure 1). However, estimates of effect sizes were inflated when only 1 of the 2 variants in high LD and having the same effect direction was included in the model during MCMC simulation (e.g. SNVs 7 and 10). The estimated effect size for SNV 8 (true effect size ≈ 0) was also inflated because it is in high LD with SNV 5 (true effect size <0). In addition, for many causal SNVs there was a high posterior exclusion probability (the proportion of times the variant was not included in the model), suggesting that some beta estimates were based on a small number of MCMC runs.

## Discussion

We developed a novel Bayesian model for analysis of multiple longitudinal traits and multi-variant models in family genetic studies. This is a significant advance because previous studies have looked at bivariate models, longitudinal data, or multi-SNV models separately. For the first time, we have developed an analytic approach that can incorporate all of these issues jointly. The model considers bivariate random family and random subject effects to account for correlation between outcomes, family members, and repeated measures of the same individual. The seemingly unrelated regression technique is used to correlate the residuals of the 2 outcomes measured at the same time for the same individual. Inherent in this method is Bayesian multiplicity for the control of false positives. Using the GAW18 simulated data set, we demonstrated the feasibility of the proposed model.

Compared to the Bayesian univariate model in which the 2 outcomes are modeled separately, our method had similar power, but fewer false positives. Compared to MGA, our Bayesian bivariate approach had fewer false positives regardless of LD between causal and noise SNVs. While the noise variants in LD with our causal variants did not result in increased false positives using the Bayesian approach, given the multi-variant nature of the analysis it is possible that the LD in these noise parameters may have reduced power to detect causal effects. Although different from our proposed method in many aspects, MGA represents a standard practice for analysis of family-based genetic studies. This comparison of different approaches is important because it demonstrates that given the low false positives, the Bayesian model is potentially better than current standard practice.

Several other studies have shown improved power of joint modeling of multiple traits over univariate analyses [10-13]. However, we saw a modest reduction in false positives without much increase in power. This may be a result of the fact that although the correlation between SBP and DBP is modest $\left(r^{2}=0.55\right)$, these 2 outcomes depend on almost exactly the same set of variants; efficiency gains by utilizing a multivariate approach in this case are negligible. In contrast, large efficiency gains are expected when the outcomes depend on different covariate sets [11]. Furthermore, our model does not explicitly model the genetic relationship between individuals; instead, we used a random family effect. Although we do not expect the inclusion of a kinship coefficient matrix to influence the results, future studies should examine this further.

It is important to note that the model inference is based on computationally expensive MCMC simulation. Although we didn't use any special algorithms to efficiently sample the model space and the number of iterations was relatively small given the extremely large model space, the posterior inclusion probabilities became stable very early in the MCMC chain. This suggests that posterior inclusion probabilities can provide valid model inference. Sensitivity analyses with more optimistic priors on inclusion probabilities gave more power while controlling false positives. However, prior knowledge or evidence must support the use of this type of prior.

## Conclusions

In summary, we introduced a Bayesian model for analysis of bivariate quantitative traits measured longitudinally in family genetic studies. This method extends the previous joint modeling methods and permits simultaneous analysis of multiple traits with longitudinal data. Furthermore, this method incorporates multi-variant effects while effectively controlling the false-positive rate.

## Competing interests

The authors declare that they have no competing interests.

## Authors' contributions

LD conceived and performed the statistical analysis and drafted the manuscript. LJM contributed to the design of the statistical analysis.HH helped with statistical analysis. BGK, ESA, TBM, DWF, HH, XZ, VP, LK, and LJM helped with the writing of the manuscript. All authors read and approved the final manuscript.
